# Whole-heart contrast enhanced coronary magnetic resonance angiography using respiratory image based navigation in patients with congenital heart disease

**DOI:** 10.1186/1532-429X-17-S1-O91

**Published:** 2015-02-03

**Authors:** Miguel S Vieira, Markus Henningsson, Nathalie Dedieu, Isma Rafiq, Rene Botnar, Aaron Bell, Sujeev Mathur, Kuberan Pushparajah, Tarique Hussain, Gerald F Greil

**Affiliations:** 1 Division of Imaging Sciences & Biomedical Engineering , The Rayne Institute, King's College London, London, UK; 2 Cardiac MRI Imaging, Guy's & St Thomas NHS Foundation Trust, London, UK; 3 Congenital Cardiac MRI Imaging, Guy's & St. Thomas' Hospital/Evelina Children's Hospital, London, UK; 4 Paediatric Cardiology, Evelina London Children's Hospital, London, UK

## Background

Despite ongoing advances in magnetic resonance (MR) sequence development, coronary imaging in paediatric patients remains challenging. MR imaging of the coronary arteries can be difficult due to high heart rates (HR), small diameter of the coronary arteries, and poor contrast between the blood pool and extravascular structures. The use of higher relaxivity contrast agents has been shown to improve coronary imaging in adult patients significantly, but no data is available in paediatric patients.

## Methods

Children over 2 years-old with a clinically indicated MR study, were prospectively enrolled between September 2013 and August 2014.

Patients were imaged under general anaesthesia with an ECG-triggered, free breathing whole-heart 3D balanced steady-state free precession (bSSFP) sequence, using a respiratory image based navigator tracking the heart motion and a T2 preparation prepulse (sequence A). Gadobenate dimeglumine (0.2mL per kilogram of body weight) was then administered as a single bolus for a time-resolved 3D MR angiography. Five minutes after contrast administration, a single-shot Look-Locker pulse sequence was acquired to determine the optimal inversion time to null the myocardium. Sequence B was adapted to the reduced T1 relaxation time of the blood pool, including an inversion prepulse to null signal from myocardium. Coronary reformatting and quantitative analysis of vessel length, diameter and sharpness was performed using dedicated software ("Soap-Bubble").

## Results

Twenty-nine consecutive paediatric patients were included (21 male; 8 female; mean age=5.28 ± 3.4 years). There was no significant difference in the HR during both sequences acquisitions (mean HR of 78 ± 14.3bpm sequence A vs 78 ± 14.4bpm sequence B; p=0.698). Mean vessel length was 4.59 ± 2.51mm (A) vs 4.52 ± 2.69mm (B) (p=0.78) for a mean vessel diameter (first 4cm) of 2.14 ± 0.38mm (A) vs 2.08 ± 0.42mm (B) (p=0.091). The use of an inversion recovery bSSFP 3D sequence with gadobenate dimeglumine resulted in improved SNR (p<0.005), CNR (p=0.005), and vessel wall sharpness (p<0.005). There was no correlation between HR during acquisition and vessel sharpness (p=0.335).

## Conclusions

The use of gadobenate dimeglumine with a specific sequence design results in improved coronary imaging quality even in small children with high HR. This may allow to replace invasive cardiac catheterization for diagnostic coronary imaging and preoperative planning in these patients.

## Funding

MSV received a research grant from Bracco® (Bracco Diagnostics. Inc., Italy). Institution received contrast agents and imaging time from Bracco® (Bracco Diagnostics. Inc., Italy).

**Figure 1:**
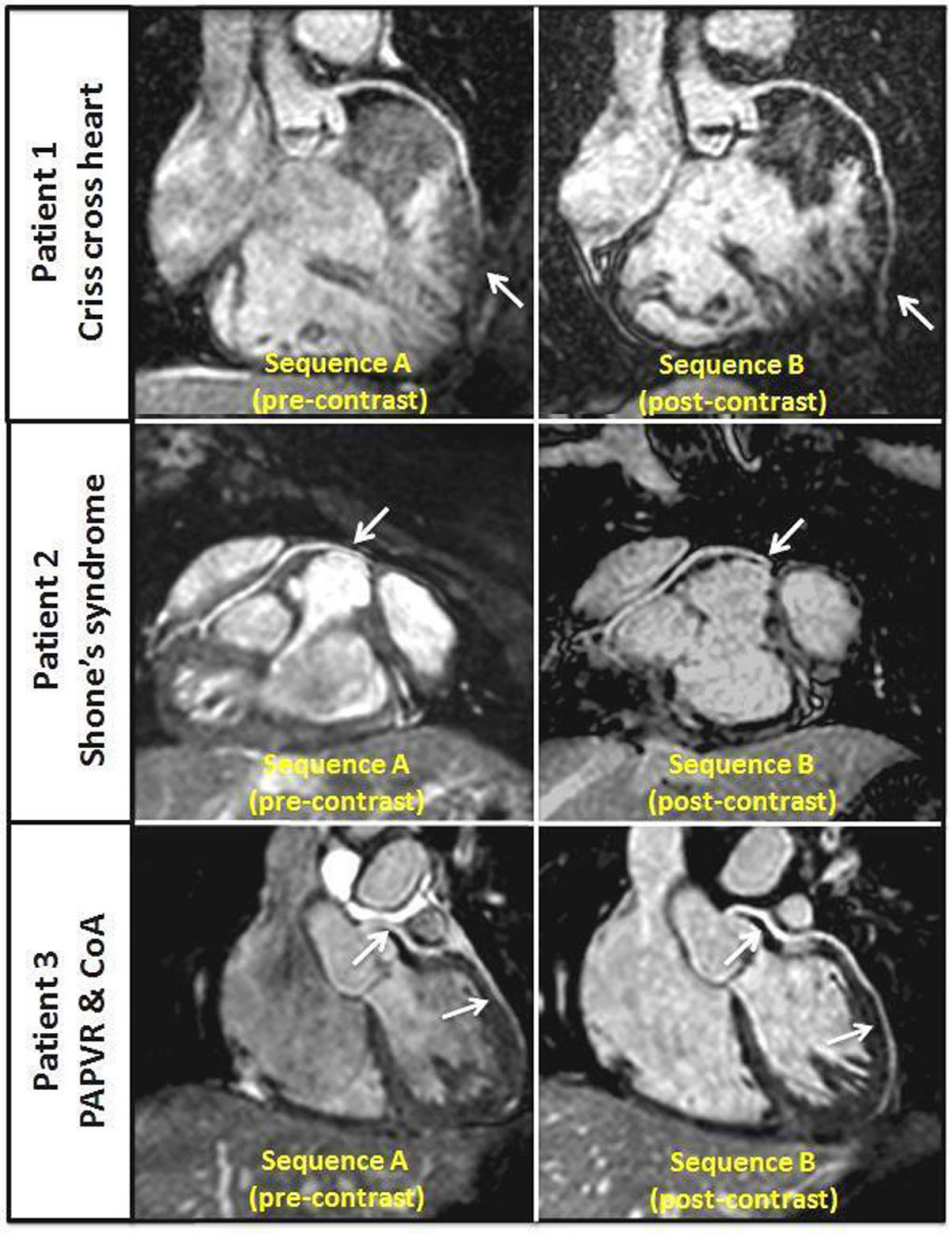
Whole-heart 3D steady-state free precession coronary magnetic resonance images from three patients reformatted using Soap-Bubble software. Left-hand panels - sequence A (pre-contrast). Right-hand panels - sequence B (post-contrast). Arrows point to coronary segments with improved visualisation after contrast. PAPVR, Partial anomalous pulmonary venous return. CoA, aortic coarctation.

